# Impact of Prior Q-Wave Myocardial Infarction in Transcatheter Aortic Valve Replacement Patients With Reduced Ejection Fraction

**DOI:** 10.1016/j.shj.2025.100731

**Published:** 2025-09-26

**Authors:** Siddhartha Mengi, Marina Urena, Gabriela Veiga-Fernandez, Alberto Alperi, Luis Nombela-Franco, Victoria Vilalta, Ander Regueiro, Jules Mesnier, Victor Fradejas-Sastre, Pablo Avanzas, Ricardo Ortiz Lozada, Elena de Oliveira-Cañedo, Giovanni Occhipinti, Pedro Cepas-Guillén, Marisa Avvedimento, Josep Rodés-Cabau

**Affiliations:** a Quebec Heart & Lung Institute, Laval University, Quebec City, Quebec, Canada; b Department of Cardiology, Hospital Bichat Claude-Bernard, Paris, France; c Cardiology Department, Hospital Universitario Marqués de Valdecilla, Instituto de Investigación Valdecilla (IDIVAL), Santander, Spain; d Department of Cardiology, Hospital Universitario Central de Asturias, Oviedo, Spain; e Cardiovascular Institute, Hospital Clínico San Carlos, Instituto de Investigación Sanitaria Hospital Clínico San Carlos (IdISSC), Madrid, Spain; f Department of Cardiology, Hospital Universitari Germans Trias i Pujol, Badalona, Spain; g Department of Cardiology, Instituto Clínic Cardiovascular, Hospital Clínic, and Institut d'Investigacions Biomèdiques August Pi i Sunyer (IDIBAPS), Barcelona, Spain

**Keywords:** CAD, LVEF, Q-wave MI, TAVR

## Abstract

**Background:**

Coronary artery disease is prevalent in transcatheter aortic valve replacement (TAVR) patients, but the specific impact of prior Q-wave myocardial infarction (QWMI), a marker of transmural infarction, remains underexplored. This study evaluated the clinical impact of QWMI in TAVR patients with left ventricular ejection fraction (LVEF) ​< ​50%.

**Methods:**

Multicenter study including 1172 consecutive patients undergoing TAVR with contemporary devices, stratified according to prior QWMI. The primary outcome was all-cause mortality or heart failure hospitalization (HFH) over a median follow-up of 3 (1-4) years. Secondary endpoints included changes in LVEF and independent predictors of adverse outcomes.

**Results:**

Prior QWMI was present in 106 (9.0%) patients. Those with QWMI were younger (77.3 vs 80.6 years; *p* ​< ​0.001) and had lower baseline LVEF (36.3 vs. 38.3%; *p* ​= ​0.048). Unadjusted analysis showed higher all-cause mortality (49.1 vs 31.7%; *p* ​< ​0.001) and combined death or HFH (54.8 vs 36.7%; *p* ​< ​0.001). LVEF improvement at 30 days was attenuated in QWMI patients, particularly with anterior MI. After adjustment, QWMI was not independently associated with mortality or combined death/HFH. Chronic kidney disease (hazard ratio: 1.26; *p* ​= ​0.042) and permanent atrial fibrillation (hazard ratio: 1.30; *p* ​= ​0.013) were independent predictors of adverse outcomes.

**Conclusions:**

Up to 1 out of 10 TAVR patients with reduced LVEF had prior QWMI, which was associated with impaired LVEF recovery, especially in those with anterior QWMI, and worse clinical outcomes at 3-year follow-up, largely driven by comorbidities. These findings underscore the importance of advanced preprocedural imaging, tailored therapeutic strategies, and integrated multidisciplinary care to enhance outcomes in this high-risk TAVR population.

## Highlights

• Prior Q-wave MI was linked to impaired LVEF recovery after TAVR.
• Anterior QWMI patients had the least improvement in post-TAVR LVEF.
• QWMI was not an independent predictor of mortality or HF hospitalization.
• CKD and atrial fibrillation were stronger outcome predictors post-TAVR.

## Graphical abstract

**Figure:**
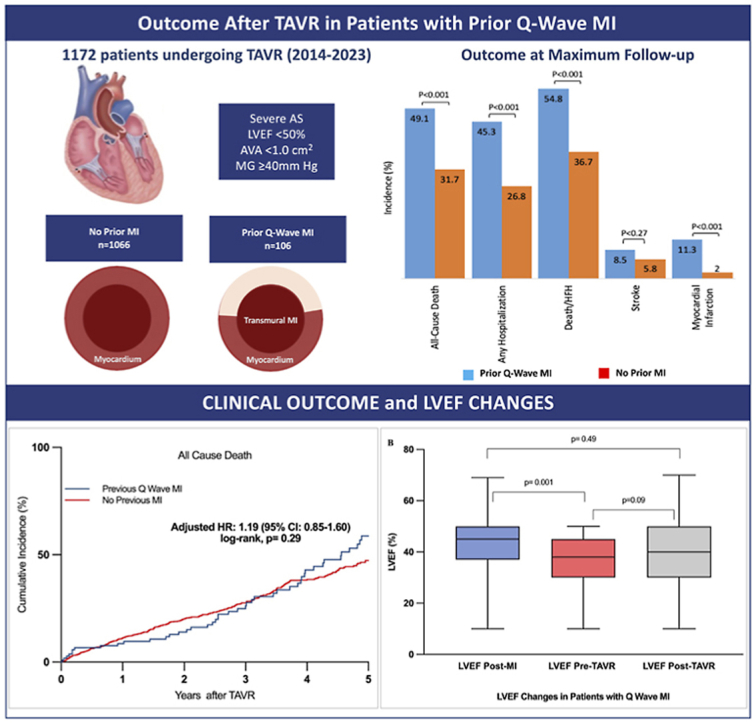

## Introduction

Transcatheter aortic valve replacement (TAVR) has transformed the management of severe aortic stenosis (AS) for patients across the surgical risk spectrum.^1^, ^2^, ^3^ Severe AS imposes hemodynamic stress on the left ventricle (LV) through pressure overload, leading to an increased risk of LV dysfunction and heart failure (HF) if untreated.^4^ However, the extent of clinical benefit from TAVR may be influenced by underlying myocardial health and comorbidities, particularly coronary artery disease (CAD) and prior myocardial infarction (MI). Studies report that CAD is present in 30%-75% of patients with severe AS, and prior MI is a frequent comorbidity in this group.^5^, ^6^, ^7^ Within this population, patients with a history of Q-wave MI (QWMI) represent a particularly vulnerable subgroup, in whom the irreversible LV damage may limit the potential benefits of TAVR.

QWMI, characterized by full-thickness (transmural) myocardial necrosis, leads to irreversible LV remodeling and scar formation.^8^ This process reduces LV ejection fraction (LVEF), increases the risk of HF, and is associated with poorer clinical outcomes. In the setting of severe AS, the combination of chronic pressure overload and prior infarcted myocardium may severely restrict the capacity for LV functional recovery following TAVR, given the combined burden of ischemic and valvular pathology. While CAD is a well-established risk factor in TAVR, the specific impact of prior QWMI on mortality and LVEF improvement in patients with severe AS has not been previously explored. Furthermore, patients with prior QWMI often present with a higher burden of comorbidities, such as diabetes and chronic kidney disease (CKD), which may further compound adverse outcomes.^9^ Current valvular guidelines also do not offer specific recommendations for risk stratification or management in this subgroup, underscoring the need for more robust evidence.^10^^,^^11^ Thus, this study aimed to evaluate the impact of prior QWMI on clinical and echocardiographic outcomes in patients with severe AS and LV dysfunction undergoing TAVR, compared to patients without prior MI.

## Methods

### Study Design

This multicenter cohort study included patients with severe AS and LVEF <50% who underwent transarterial TAVR between 2014 and 2023 across 7 centers. Severe AS was defined as an aortic valve area <1 ​cm^2^ or a mean transvalvular gradient ≥40 ​mmHg. Patients were stratified into 2 groups based on the presence or absence of prior QWMI. Indications for TAVR, device type, and procedural approach were assessed by the heart team at each center, based on an extensive clinical and anatomic preoperative assessment that included CT evaluation for all patients before the procedure. The study was performed in accordance with the ethics committee of each center, and all patients provided written informed consents for the procedures.

### Data Collection and Clinical Endpoints

Baseline clinical, echocardiographic, procedural, and follow-up data were prospectively collected using a standardized TAVR database at each center. Follow-up data were obtained via clinical visits, telephone interviews, or medical record reviews at 30 days and annually thereafter up to a median of 3 years (interquartile range [IQR]: 1–4 years). All endpoints were defined according to the Valve Academic Research Consortium-3 criteria.^12^ The primary outcome was all-cause mortality or heart failure hospitalization (HFH) at maximum follow-up. Secondary outcomes included all periprocedural and maximum follow-up adverse events, as well as changes in LVEF from baseline to 30-day follow-up. Echocardiographic data including LVEF, transaortic gradients, and aortic valve area were site-reported, following American Society of Echocardiography guidelines.^13^ Postinfarction LVEF values were retrospectively available in 88 patients (83%) with prior QWMI. These were extracted from clinical records and assessed assessed by transthoracic echocardiography or left ventriculogram. However, the timing and measurement protocols were not standardized. In contrast, pre-TAVR and post-TAVR LVEFs were prospectively acquired using consistent transthoracic echocardiography methodology at all sites.

### Q-Wave MI Definition

QWMI was defined as a history of ST-elevation MI with pathological Q-waves on baseline electrocardiography, as per the Fourth Universal Definition of MI.^9^ Pathological Q-waves were identified and confirmed by local cardiologists as any Q wave in leads V2–V3 > 0.02 ​seconds or a QS complex in these leads. Additionally, a Q wave ≥0.03 ​seconds and ≥1 ​mm deep, or a QS complex in leads I, II, aVL, aVF, or V4–V6 in any 2 contiguous leads were considered significant. Furthermore, an R wave >0.04 ​seconds in V1–V2 with an R/S ratio >1 and a concordant positive T wave, in the absence of a conduction defect, also defined a pathological Q wave.

### Statistical Analysis

Categorical variables were presented as n (%) and continuous variables as mean ​± ​standard deviation or median (IQR). Group comparisons were performed with the chi-square test for categorical variables. For numerical variables, Student’s *t*-test or Kruskal-Wallis test were used for normally distributed continuous variables, and Wilcoxon rank-sum test for non-normal data. Kaplan-Meier survival curves were constructed for time-to-event outcomes (all-cause mortality, combined death/HFH), with log-rank tests for group differences. Univariable and multivariable Cox proportional hazards models assessed predictors of all-cause mortality, reporting hazard ratios (HRs) with 95% confidence intervals (CIs). Variables with *p* ​< ​0.10 in univariable analysis (age, sex, CKD, permanent atrial fibrillation [AF], chronic obstructive pulmonary disease, pacemaker implantation/stroke at 30 days) were included in the multivariable model, adjusted for center variability using a hierarchical approach. The proportional hazards assumption was verified using Schoenfeld residuals. A two-tailed *p* ​< ​0.05 denoted statistical significance. Analyses were performed using Stata (V14.0; StataCorp LP, College Station, Texas, USA) and Prism (V10.1.1; GraphPad Software, Boston, Massachusetts, USA).

## Results

The study included 1172 consecutive patients, stratified into those with prior QWMI (n ​= ​106, 9.0%) and those without prior MI (n ​= ​1,066, 91%). The mean age of the study population was 80.3 ​± ​7.6 years, with 36% of women, and a mean Society of Thoracic Surgeons score of 6.2 ​± ​5.1. Baseline clinical and echocardiographic characteristics are shown in Table 1. Patients with QWMI had a higher burden of cardiovascular comorbidities, including prior percutaneous coronary intervention (64.2 vs 26.4%; *p* ​< ​0.001), coronary artery bypass grafting (42.5 vs 10.7%; *p* ​< ​0.001), and peripheral artery disease (29.3 vs 16.8%; *p* ​= ​0.002). Baseline LVEF was lower in QWMI patients (36.3% ​± ​10.2% vs 38.3% ​± ​8.6%; *p* ​= ​0.048), with lower peak (58.0 ​± ​19.6 ​mmHg vs 66.2 ​± ​24.4 ​mmHg; *p* ​= ​0.001) and mean (34.8 ​± ​13.0 ​mmHg vs 39.7 ​± ​15.7 ​mmHg; *p* ​= ​0.002) transaortic gradients.

**Table 1:** Clinical and echocardiographic characteristics, overall and according to Q-wave MI status

	Total	Previous Q-Wave MI	No previous MI	*p* value
	N ​= ​1172	N ​= ​106	N ​= ​1066	
Age, y	80.3 ​± ​7.6	77.3 ​± ​9.0	80.6 ​± ​7.4	**<0.001**
Female sex	422 (36.0%)	15 (14.2%)	407 (38.2%)	**<0.001**
BMI, kg/m2	26.7 ​± ​5.2	26.3 ​± ​4.6	26.7 ​± ​5.3	0.47
NYHA III-IV	804 (68.6%)	68 (64.2%)	736 (69.0%)	0.30
Diabetes mellitus	431 (36.8%)	41 (38.7%)	390 (36.7%)	0.69
Hypertension	963 (82.2%)	83 (78.3%)	880 (82.6%)	0.28
Previous CAD	521 (44.5%)	106 (100.0%)	415 (38.9%)	**<0.001**
Previous PCI	349 (29.8%)	68 (64.2%)	281 (26.4%)	**<0.001**
Previous CABG	159 (13.6%)	45 (42.5%)	114 (10.7%)	**<0.001**
Peripheral artery disease	210 (17.9%)	31 (29.3%)	179 (16.8%)	**0.002**
Previous stroke	131 (11.2%)	13 (12.3%)	118 (11.1%)	0.71
Chronic obstructive pulmonary disease	286 (24.4%)	19 (18.1%)	267 (25.0%)	0.11
Permanent AF	510 (43.5%)	35 (33.0%)	475 (44.6%)	**0.022**
Previous heart failure	1141 (97.4%)	99 (93.4%)	1042 (97.7%)	**0.008**
Prior pacemaker	198 (16.9%)	18 (17.0%)	180 (16.9%)	0.98
Chronic kidney disease (eGFR<60)	683 (58.3%)	55 (51.9%)	628 (58.9%)	0.16
Hemoglobin levels	12.2 (10.9-13.4)	12.4 (11.0-13.8)	12.2 (10.9-13.3)	0.50
EuroScore II	9.1 ​± ​8.3	8.7 ​± ​7.4	9.1 ​± ​8.4	0.65
STS-PROM score	6.2 ​± ​5.1	6.4 ​± ​6.1	6.2 ​± ​5.1	0.67
Echocardiographic findings at baseline				
LVEF, %	37.8 ​± ​8.7	36.3 ​± ​10.2	38.3 ​± ​8.6	**0.048**
Peak transaortic gradient, mmHg	65.4 ​± ​24.1	58.0 ​± ​19.6	66.2 ​± ​24.4	**0.001**
Mean transaortic gradient, mmHg	39.3 ​± ​15.6	34.8 ​± ​13.0	39.7 ​± ​15.7	**0.002**
Aortic valve area, cm^2^	0.7 ​± ​0.3	0.8 ​± ​0.2	0.7 ​± ​0.3	**0.004**
Moderate or severe AR	100 (8.5%)	12 (11.3%)	88 (8.3%)	0.28
Moderate or severe MR	50 (4.2%)	4 (3.8%)	46 (4.4%)	0.79
PASP, mmHg	35.2 ​± ​15.5	35.0 ​± ​16.5	35.7 ​± ​21.9	0.94
Medications				
Beta-blocker	707 (60.3%)	83 (78.4%)	624 (58.6%)	**0.009**
ACE inhibitor	587 (50.1%)	68 (64.1%)	519 (48.6%)	**0.049**
Angiotensin II receptor blocker	70 (6.0%)	11 (10.3%)	59 (5.5%)	0.19
Mineralocorticoid receptor antagonist	239 (20.4%)	22 (20.7%)	217 (20.4%)	0.98
Loop/thiazide-like diuretics	797 (68.0%)	73 (68.8%)	724 (67.9%)	0.91
Sacubitril/Valsartan	84 (7.2%)	7 (6.6%)	77 (7.2%)	0.79
SGLT-2 inhibitor	37 (3.2%)	7 (6.6%)	30 (2.8%)	0.26

Values are mean ​± ​SD, median (IQR) or n (%).

Abbreviations: ACE, angiotensin-converting enzyme inhibitor; AF, atrial fibrillation; AR, aortic regurgitation; BMI, body mass index; CABG, coronary artery bypass graft; CAD, coronary artery disease; eGFR, estimated glomerular filtration rate; EuroSCORE, European System for Cardiac Operative Risk Evaluation; LVEF, left ventricular ejection fraction; MI, myocardial infarction; MR, mitral regurgitation; NYHA, New York Heart Association; PASP, pulmonary artery systolic pressure; PCI, percutaneous coronary intervention; SGLT-2, sodium-glucose cotransporter-2; STS-PROM, Society of Thoracic Surgeons Predicted risk of mortality; MI, myocardial infarction.

### Procedural and Clinical Outcomes

QWMI patients less commonly underwent transfemoral TAVR (75.5 vs 93.2%; *p* ​< ​0.001), with greater use of transcarotid (8.5 vs 1.6%) and subclavian (16.0 vs 5.3%) approaches (Table 2). Clinical and echocardiographic outcomes at 30 days are outlined in Table 3. At 30 days, QWMI patients had higher rates of MI (1.9 vs 0.3%; *p* ​= ​0.016) and lower post-TAVR LVEF (40.3% ​± ​12.6% vs 42.6% ​± ​11.9%; *p* ​= ​0.029), but similar rates of death (1.9 vs 2.4%; *p* ​= ​0.72), stroke (2.8 vs 1.8%; *p* ​= ​0.45), and major vascular complications (9.5 vs 5.3%; *p* ​= ​0.054).

**Table 2:** Procedural characteristics overall and according to Q-wave MI status

	Total	Previous Q wave MI	No previous MI	*p* value
	N ​= ​1172	N ​= ​106	N ​= ​1066	
Approach				**<0.001**
Transfemoral	1073 (91.6%)	80 (75.5%)	993 (93.2%)	
Transcarotid	26 (2.2%)	9 (8.5%)	17 (1.6%)	
Subclavicular	73 (6.2%)	17 (16.0%)	56 (5.3%)	
Secondary access				0.55
Femoral	454 (38.8%)	44 (41.6%)	411 (38.5%)	
Radial	718 (61.2%)	62 (58.4%)	656 (61.5%)	
Prostheses type				0.11
Balloon-expandable	726 (62.0%)	75 (70.8%)	651 (61.1%)	
Self-expandable	446 (38.1%)	31 (29.2%)	415 (38.9%)	
Predilatation	333 (28.4%)	29 (27.4%)	304 (28.5%)	0.80
Postdilatation	165 (14.1%)	14 (13.3%)	151 (14.2%)	0.82
Valve-in-valve procedure	87 (7.5%)	4 (3.8%)	83 (7.8%)	0.13

Abbreviation: MI, myocardial infarction.

Values are mean ​± ​SD or n (%).

**Table 3:** 30-day clinical and echocardiographic outcomes

	Total	Previous Q wave MI	No previous MI	*p* value
	N ​= ​1172	N ​= ​106	N ​= ​1066	
30-d outcomes				
Death	28 (2.4%)	2 (1.9%)	26 (2.4%)	0.72
All stroke	22 (1.9%)	3 (2.8%)	19 (1.8%)	0.45
Myocardial infarction	5 (0.4%)	2 (1.9%)	3 (0.3%)	**0.016**
Major vascular complication	66 (5.7%)	10 (9.5%)	56 (5.3%)	0.054
Bleeding event according to VARC-3				0.96
Type 1	73 (28.9%)	8 (25.8%)	65 (29.3%)	
Type 2	148 (58.5%)	19 (61.3%)	129 (58.1%)	
Type 3	31 (12.3%)	4 (12.9%)	27 (12.2%)	
Type 4	1 (0.4%)	0 (0.0%)	1 (0.5%)	
Acute kidney injury	116 (9.9%)	8 (7.5%)	108 (10.1%)	0.39
Permanent pacemaker implantation	175 (14.9%)	19 (17.9%)	156 (14.6%)	0.36
Heart failure hospitalization	3 (0.2%)	2 (1.9%)	1 (0.1%)	0.61
Echocardiography				
LVEF, %	42.5 ​± ​11.8	40.3 ​± ​12.6	42.6 ​± ​11.9	**0.029**
Peak transaortic gradient, mmHg	18.3 ​± ​8.3	18.2 ​± ​8.8	18.3 ​± ​8.2	0.96
Mean transaortic gradient, mmHg	9.8 ​± ​4.3	9.6 ​± ​4.6	10.0 ​± ​4.2	0.90
Aortic valve area, cm^2^	1.7 ​± ​0.5	1.8 ​± ​0.6	1.7 ​± ​0.5	**0.017**
Moderate or severe AR	10 (0.8%)	2 (1.9%)	8 (0.7%)	0.25

Values are mean ​± ​SD or n (%).

Abbreviations: AR, aortic regurgitation; LVEF, left ventricular ejection fraction; MI, myocardial infarction; VARC-3, Valve Academic Research Consortium-3.

**Figure 1:**
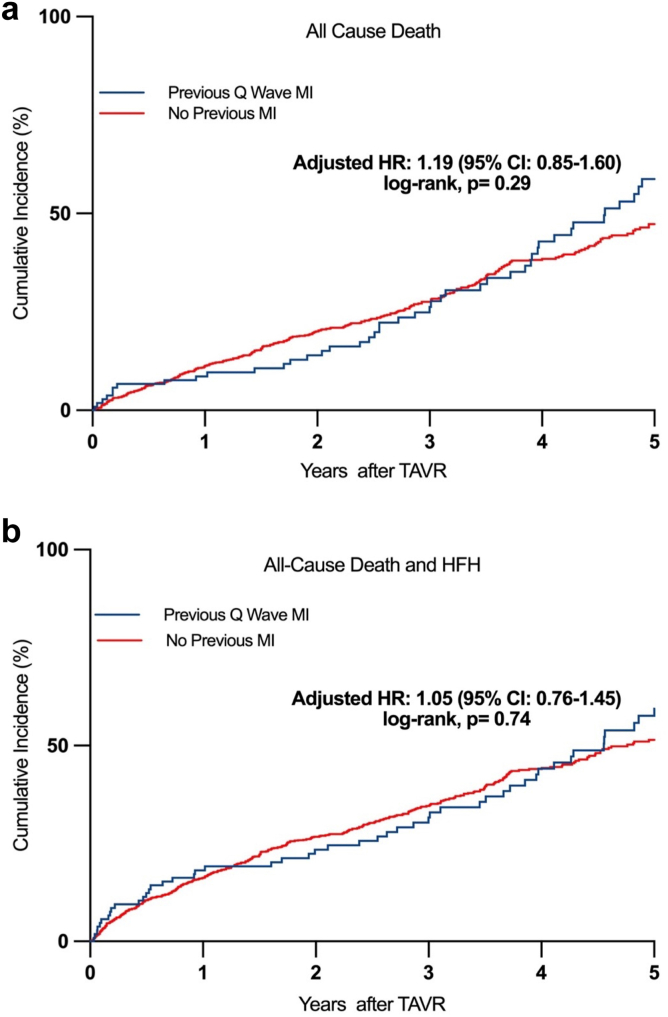
Kaplan-Meier estimates of **(A)** all-cause mortality and **(B)** combined all-cause mortality or HFH according to the presence of QWMI. Hazard ratio adjusted for age, female sex, chronic obstructive pulmonary disease, comorbidities (chronic kidney disease, diabetes), and valve type. Abbreviations: CI, confidence interval; HFH, heart failure hospitalization; HR, hazard ratio; MI, myocardial infarction; TAVR, transcatheter aortic valve replacement.

At maximum follow-up (median follow-up: 3 years [IQR: 1-4 years]), a total of 390 patients (33.3%) died, and QWMI patients exhibited higher all-cause mortality (49.1 vs 31.7%; *p* ​< ​0.001), MI (11.3 vs 2.0%; *p* ​< ​0.001), any hospitalization (45.3 vs 26.8%; *p* ​< ​0.001), and combined death and HFH (54.8 vs 36.7%; *p* ​< ​0.001) (Table 4). Cardiovascular mortality (48.1 vs 48.2%; *p* ​= ​0.99) was similar. The baseline characteristics, procedural, and clinical outcomes among QWMI vs. non-QWMI are presented in Supplemental Tables S1 to S4. The Kaplan–Meier survival curves for all-cause mortality and the composite endpoint of all-cause mortality or HFH stratified by prior MI status are presented in Figure 1. After adjustment for baseline characteristics, prior QWMI was not independently associated with all-cause mortality (adjusted HR: 1.19; 95% CI: 0.85–1.60; p ​= ​0.29) or the composite of death and HFH (adjusted HR: 1.05; 95% CI: 0.76–1.45; p ​= ​0.74).

**Table 4:** Clinical outcomes at follow-up, overall and according to the presence of prior Q-wave myocardial infarction, including adjusted risk of adverse outcomes

	Total	Previous Q wave MI	No previous MI	*p* value	Adjusted HR∗ (95% CI)	*p* value
	N ​= ​1172	N ​= ​106	N ​= ​1066			
All-cause death	390 (33.3%)	52 (49.1%)	338 (31.7%)	**<0.001**	1.19 (0.85 - 1.60)	0.29
CV death	188 (48.2%)	25 (48.1%)	163 (48.2%)	0.99	1.21 (0.74 - 1.97)	0.44
All stroke	71 (6.1%)	9 (8.5%)	62 (5.8%)	0.27	1.06 (0.49 - 2.32)	0.87
Myocardial infarction	33 (2.8%)	12 (11.3%)	21 (2.0%)	**<0.001**	2.73 (1.24 – 5.99)	**0.012**
Endocarditis	13 (1.1%)	2 (1.9%)	11 (1.0%)	0.42	1.04 (0.21 - 5.06)	0.96
Any hospitalization	333 (28.4%)	48 (45.3%)	285 (26.8%)	**<0.001**	1.14 (0.81 - 1.62)	0.43
Heart failure hospitalization (HFH)	139 (11.9%)	17 (16.0%)	122 (11.4%)	0.16	0.93 (0.49 - 1.76)	0.83
Combined death or HFH	449 (38.3%)	58 (54.8%)	391 (36.7%)	**<0.001**	1.05 (0.76 - 1.45)	0.74

Values are mean ​± ​SD or n (%).

Abbreviations: CI, confidence interval; CV, cardiovascular; HR, hazard ratio; MI, myocardial infarction.

∗ Adjusted for age, female sex, Society of Thoracic Surgeons Score, comorbidities (chronic kidney disease, diabetes), and valve type.

### Echocardiographic Findings and LVEF Recovery

**Figure 2:**
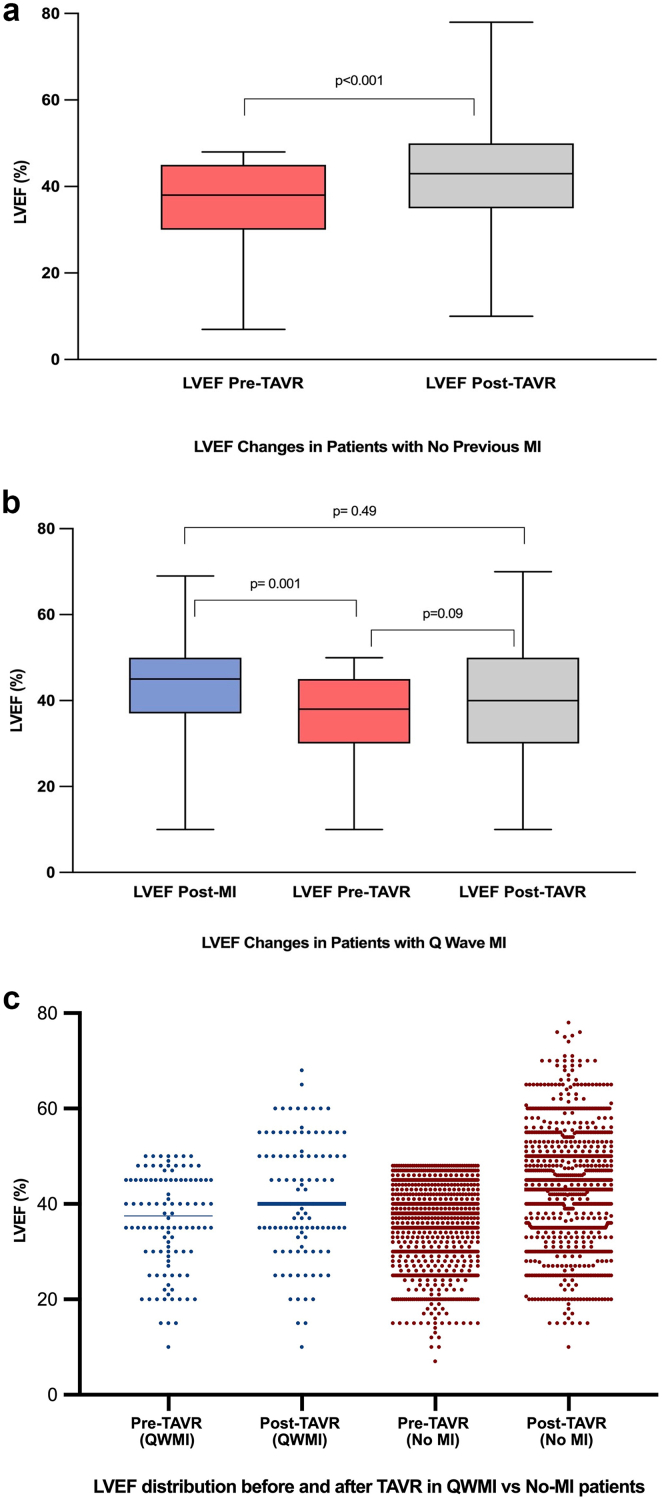
**LVEF changes in the 2 groups at baseline and 30 days after TAVR. (A)** Patients with no previous MI. **(B)** Patients with previous Q-wave MI. **(C)** LVEF distribution before and after TAVR in Q-wave MI vs. no-MI patients. Abbreviations: LVEF, left ventricular ejection fraction; MI, myocardial infarction; QWMI, Q-wave myocardial infarction; TAVR, transcatheter aortic valve replacement.

**Figure 3:**
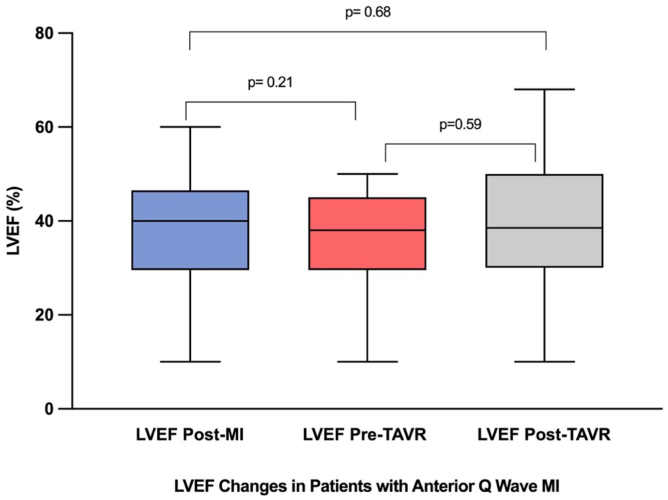
**LVEF changes in patients with anterior Q-wave myocardial infarction.** Subgroup analysis of anterior QWMI patients demonstrated no significant change in LVEF between post-MI, pre-TAVR, and post-TAVR time points, with no overall improvement over time. Abbreviations: LVEF, left ventricular ejection fraction; MI, myocardial infarction; QWMI, Q-wave myocardial infarction; TAVR, transcatheter aortic valve replacement.

Baseline LVEF was lower in the QWMI group compared with the no-MI group (36.3 ​± ​10.2 vs 38.3 ​± ​8.6; *p* ​= ​0.048). LVEF at 30 days was available for 97.6% of patients (1144/1172; 98.1% [104/106] in QWMI, 97.6% [1040/1066] in no-MI), with missing data attributable exclusively to 30-day mortality (2.4% overall; 1.9% in QWMI, 2.4% in no-MI). As illustrated in Figure 2, patients without prior MI experienced a significant improvement in LVEF following TAVR (*p* ​< ​0.001) (Panel A). In contrast, patients with prior QWMI demonstrated blunted LVEF recovery. While LVEF declined from pre-MI levels to pre-TAVR values (*p* ​= ​0.001), the subsequent increase in LVEF post-TAVR did not reach statistical significance (*p* ​= ​0.09), and the overall difference between post-QWMI LVEF and post-TAVR LVEF in this group remained nonsignificant (*p* ​= ​0.49) (Panel B). Panel C further illustrates the individual-level distribution of LVEF values at baseline and 30 days post-TAVR in both groups. Subgroup analysis showed that patients with anterior QWMI demonstrated no significant change in LVEF between post-MI, pre-TAVR, and post-TAVR time points (*p* ​= ​0.21 and *p* ​= ​0.59), with no overall improvement over time (*p* ​= ​0.68), indicating minimal recovery of LV function in this subgroup despite TAVR (Figure 3). A sensitivity analysis comparing anterior versus non-anterior QWMI subgroups is summarized in Supplemental Table S4.

### Predictors of Adverse Outcomes

In multivariable Cox models adjusted for age, sex, chronic obstructive pulmonary disease, CKD, diabetes, and valve type, QWMI was not independently associated with all-cause mortality (HR: 1.19; 95% CI: 0.85-1.60; *p* ​= ​0.29) or combined death or HFH (HR: 1.05; 95% CI: 0.76-1.45; *p* ​= ​0.74) (Table 4). Independent predictors of all-cause mortality included male sex (HR: 1.58; 95% CI: 1.26-2.0; *p* ​< ​ 0.001), CKD (HR: 1.36; 95% CI: 1.11-1.66; *p* ​= ​0.002), and permanent AF (HR: 1.24; 95% CI: 1.00-1.53; *p* ​= ​0.043) (Table 5).

**Table 5:** Predictors of mortality or HF hospitalization post-TAVR

	Univariable model	*p* value	Multivariable model	*p* value
	HR (95% CI)		HR (95% CI)	
Age	1.01 (1.0 - 1.02)	**0.05**	1.01 (0.99 - 1.02)	0.09
Female sex	0.69 (0.57 - 0.85)	**0.001**	0.68 (0.55 - 0.85)	**<0.001**
NYHA III-IV	1.04 (0.85 - 1.27)	0.68		
Chronic kidney disease	1.41 (1.16 - 1.72)	**<0.001**	1.36 (1.11 - 1.66)	**0.002**
Diabetes mellitus	1.14 (0.94 - 1.38)	0.16		
Permanent AF	1.34 (1.09 - 1.65)	**0.005**	1.24 (1.01 – 1.53)	**0.043**
COPD	1.12 (0.92 - 1.39)	0.15	1.08 (0.87 – 1.35)	0.76
Hemoglobin levels∗	1.14 (0.84 - 1.81)	0.59		
Previous HF	1.09 (0.60 – 1.99)	0.76		
Valve type (balloon vs. self-expandable)	1.05 (0.87 - 1.27)	0.58		
Q-wave MI	0.97 (0.73 - 1.30)	0.84		
LVEF pre-TAVR (per 5% decrease)	0.97 (0.70 - 1.34)	0.87		
Pacemaker ≤30 ​d	1.25 (1.00 -1.61)	**0.05**	1.16 (0.91 - 1.51)	0.23
Stroke ≤30 ​d	1.79 (1.01 – 3.19)	**0.04**	1.58 (0.83 – 2.99)	0.16

Abbreviations: AF, ​atrial fibrillation; CI, confidence interval; COPD, chronic obstructive pulmonary disease; HF, heart failure; HR, hazard ratio; LVEF, left ventricular ejection fraction; MI, myocardial infarction; NYHA, New York Heart Association; TAVR, transcatheter aortic valve replacement.

∗ For each decrease of 1g/dl in hemoglobin levels.

## Discussion

To the best of our knowledge, this is the first study to date evaluating the impact of prior QWMI on outcomes after TAVR in patients with severe AS and reduced LVEF. Our major findings were as follows: 1) 1 out of 10 patients with severe AS and reduced left ventricular function had a history of prior QWMI; 2) patients with QWMI demonstrated significantly higher unadjusted rates of all-cause mortality and combined mortality/HFH rates compared to those without prior MI, however, after adjustment, prior QWMI was not independently associated with increased mortality or combined mortality/HFH; 3) improvement in LVEF at 30 days post-TAVR was significantly less in patients with prior QWMI compared to those without previous MI, suggesting a potential limitation in myocardial recovery in this subgroup; 4) among patients with prior QWMI, those with anterior infarcts had the least improvement in LVEF after TAVR, underscoring the importance of infarct location in functional recovery **(Graphical Abstract)**.

The prognostic impact of CAD in patients undergoing TAVR has long been debated, with prevalence rates ranging from 30%–75% across registries and randomized trials, yet the specific implications of prior MI, particularly QWMI, remain underexplored.^1^, ^2^, ^3^^,^^7^^,^^14^, ^15^, ^16^ This study is the first to specifically evaluate the prognostic impact of QWMI in TAVR patients, distinguishing it from prior studies that broadly assessed CAD or nonspecific MI.^17^, ^18^, ^19^ Previous observational studies and meta-analyses have yielded conflicting conclusions, some reporting adverse associations between CAD and clinical outcomes post-TAVR, others finding no independent effect after multivariable adjustment.^14^^,^^20^^,^^21^ Much of this discrepancy likely stems from heterogeneity in CAD definitions, severity grading, and revascularization strategies, as well as variability in endpoints and follow-up duration.

The lack of independent association with mortality in our adjusted models contrasts with unadjusted findings, suggesting that QWMI’s prognostic impact is mainly mediated by baseline comorbidities. In accordance with our results, prior studies reported that CAD’s prognostic impact in TAVR is often confounded by baseline comorbidities like diabetes and renal dysfunction.^17^^,^^22^ However, our study’s focus on QWMI highlights a subgroup with potentially greater myocardial damage, which may explain the pronounced unadjusted differences in mortality and HFH. The absence of an independent effect suggests that factors such as CKD and permanent AF are stronger drivers of adverse outcomes post-TAVR, aligning with previous studies^23^ (Table 5). Permanent AF is well established as an independent predictor of mortality and HFH, likely due to its frequent association with multiple noncardiac comorbidities that directly affect prognosis.^23^ CKD, similarly, has been robustly linked to poor prognosis following TAVR, likely due to impaired metabolic reserve, susceptibility to contrast-induced nephropathy, and systemic inflammation.^24^ Collectively, these findings reinforce the need for a multidimensional approach in TAVR candidates with permanent AF and/or CKD, integrating arrhythmia control, renal protection, and comorbidity management to improve long-term outcomes.

A key finding of this study is the attenuated LVEF improvement in patients with prior QWMI compared to those without pior MI. This observation aligns with the pathophysiology of transmural infarction, where myocardial scarring leads to irreversible fibrosis, limits contractile reserve, and reverse remodeling post-TAVR.^25^^,^^26^ This is consistent with Witberg et al, who reported impaired LV recovery in TAVR patients with baseline LV dysfunction and prior ischemic injury.^27^ The smaller aortic valve area post-TAVR in the QWMI group may further contribute to persistent hemodynamic stress, potentially exacerbating myocardial dysfunction. These findings are particularly relevant given the established role of LVEF as a predictor of long-term outcomes in HF and post-TAVR populations.^4^^,^^28^ The higher 30-day MI rate in QWMI patients (1.9 vs 0.3%; *p* ​= ​0.016) may reflect ongoing coronary vulnerability, possibly exacerbated by procedural factors such as hypotension or microembolization. These observations highlight the need for targeted periprocedural strategies, such as optimized coronary revascularization or enhanced anti-ischemic therapy, in this high-risk subgroup.

QWMI causes irreversible myocardial scarring, limiting the contractile benefit of afterload reduction with TAVR and explaining the blunted LVEF recovery observed in these patients (Figure 2).^29^ In contrast, patients without prior MI benefit from reverse remodeling post-TAVR, as their LV dysfunction is primarily driven by pressure overload.^8^^,^^30^ The lack of LVEF improvement in the anterior QWMI subgroup (Figure 3) likely reflects larger infarct territories, as anterior infarctions typically involve the left anterior descending artery, resulting in extensive myocardial necrosis. This may explain the subgroup’s poor response to TAVR, as the scarred anterior wall cannot contribute to LVEF recovery postvalve replacement. These mechanisms highlight the distinct pathophysiology of QWMI in TAVR, where ischemic damage compounds valvular stress. Also, anterior QWMI may increase the risk of electrical instability, such as ventricular arrhythmias, further compromising outcomes.^31^ Furthermore, the outcome of our study could partially challenge the cardiac damage staging system by demonstrating that prior QWMI, despite contributing to LV damage (stage 1 or higher), does not independently predict worse mortality or HFH post-TAVR after risk adjustment.^32^ The attenuated LVEF improvement in QWMI patients suggests that functional outcomes, not fully captured by the cardiac damage classification system, are critical in this population. These findings highlight the need to refine the staging system to account for CAD heterogeneity, myocardial viability, and longer-term outcomes, ensuring more precise risk stratification for TAVR patients with prior MI.

The lack of an independent association between prior QWMI and short-term mortality suggests that TAVR remains a viable treatment option for patients with severe AS and history of transmural MI, even in the presence of reduced LVEF. However, the attenuated LVEF improvement in this group underscores the need for careful preprocedural assessment of myocardial viability and optimization of HF therapy. Advanced imaging modalities, such as cardiac magnetic resonance for scar burden assessment, may aid in risk stratification and guide therapeutic decisions.^33^ Additionally, the higher incidence of post-TAVR MI in the QWMI group highlights the need for aggressive coronary risk management, including optimization of anti-ischemic therapies and consideration of pre-TAVR revascularization in select cases.^34^

### Limitations

This study has several limitations. First, this was a retrospective analysis of prospectively collected data, which may introduce potential selection bias despite adjustment for multiple confounders. Second, the sample size of the QWMI group was relatively small compared with the no-MI group, which may limit statistical power for detecting differences in certain outcomes. Third, data on myocardial viability or scar burden, which could influence LVEF recovery, were not available. Fourth, the study did not account for the timing of prior MI, which may impact outcomes, as more recent MIs may confer greater risk. Lastly, post-QWMI LVEF values were missing in 18 patients and were retrospectively collected, limiting the consistency and generalizability of these data in comparisons involving early myocardial recovery.

## Conclusions

This study showed that a history of QWMI is frequent in patients with AS and reduced ventricular function undergoing TAVR. Prior QWMI was associated with worse clinical outcomes, driven by a higher comorbidity burden, but QWMI was not an independent predictor of mortality or HF hospitalization. The attenuated LVEF improvement, particularly in anterior MI patients, highlights the impact of transmural scarring on functional recovery. These findings advocate for advanced pre-TAVR imaging, individualized management, and multidisciplinary care to optimize outcomes in this high-risk subgroup. Future research should explore infarct size, myocardial viability, and targeted therapies to enhance TAVR benefits in QWMI patients.

## Ethics Statement

This research was conducted in an ethical and responisble manner in accordance with all relevant and appropriate guidelines.

## Funding

The authors have no funding to report.

## Review Statement

Josep Rodes-Cabau, Marina Urena, Luis Nombela, and Marisa Avvedimento serve on the journal's editorial board. The review of this manuscript was managed by Guest Editor Neal S. Kleiman, MD.

## Disclosure Statement

J. Rodés-Cabau reports institutional research grants and consultant/speaker fees from Edwards Lifesciences and Medtronic. The other authors had no conflicts to declare.
