# Supplementary material for: Impact of Prior Q-Wave Myocardial Infarction in Transcatheter Aortic Valve Replacement Patients With Reduced Ejection Fraction
Source: Struct Heart. 2025 Sep 26;9(11):100731. doi: 10.1016/j.shj.2025.100731 (PMC12766498; doi:10.1016/j.shj.2025.100731)
Supplement: Supplemental materials [file mmc1.pdf]

Supplemental Appendix

1. Supplementary tables

1.1 Table S1………………………………………………………….. p. 2

1.2 Table S2………………………………………………………….. p. 3

1.3 Table S3………………………………………………………….. p. 4

1.4 Table S4………………………………………………………….. p. 5

1.5 Table S5…………………………………………………………... p.6

Table S1: Clinical and Echocardiographic Characteristics According to Q Wave MI vs Non-Q Wave MI status [median follow-up =3.00 (1-4) years].

	Total	Non-Q Wave MI	Q Wave MI	p-value
	**N=287**	**N=181**	**N=106**	
**Age, y**	78.8 ± 8.3	79.6 ± 7.7	77.3 ± 9.0	**0.021**
**Female sex**	64 (22.3%)	49 (27.1%)	15 (14.2%)	**0.011**
**BMI, kg/m2**	27.0 ± 5.2	27.4 ± 5.5	26.3 ± 4.6	0.093
**NYHA III-IV**	189 (65.9%)	121 (66.9%)	68 (64.2%)	0.64
**Diabetes mellitus**	140 (48.8%)	99 (54.7%)	41 (38.7%)	**0.009**
**Hypertension**	239 (83.3%)	156 (86.2%)	83 (78.3%)	0.084
**Previous myocardial infarction**	287 (100.0%)	181 (100.0%)	106 (100.0%)	
**Previous PCI**	186 (64.8%)	118 (65.2%)	68 (64.2%)	0.86
**Previous CABG**	109 (38.0%)	64 (35.4%)	45 (42.5%)	0.23
**Peripheral artery disease**	69 (24.9%)	40 (22.5%)	29 (29.3%)	0.21
**Previous stroke**	34 (11.8%)	21 (11.6%)	13 (12.3%)	0.87
**Chronic obstructive pulmonary disease**	52 (18.2%)	33 (18.2%)	19 (18.1%)	0.98
**Permanent AF**	99 (34.5%)	64 (35.4%)	35 (33.0%)	0.69
**Previous heart failure**	261 (90.9%)	162 (89.5%)	99 (93.4%)	0.27
**Prior Pacemaker**	54 (18.8%)	36 (19.9%)	18 (17.0%)	0.54
**Chronic kidney disease (eGFR<60)**	161 (56.1%)	106 (58.6%)	55 (51.9%)	0.27
**Hemoglobin levels**	12.3 (10.8-13.5)	12.2 (10.7-13.5)	12.4 (11.0-13.8)	0.48
**EuroScore II**	9.4 ± 8.2	9.7 ± 8.6	8.7 ± 7.4	0.34
**STS-PROM score**	6.2 ± 5.2	6.0 ± 4.6	6.4 ± 6.1	0.61
Echocardiographic findings at baseline				
**LVEF, %**	37.7 ± 9.5	38.6 ± 9.1	36.3 ± 10.2	**0.050**
**Peak transaortic gradient, mmHg**	61.8 ± 21.0	64.0 ± 21.5	58.0 ± 19.6	**0.024**
**Mean transaortic gradient, mmHg**	37.0 ± 13.8	38.3 ± 14.1	34.8 ± 13.0	**0.036**
**Aortic valve area, cm^2**	0.7 (0.2)	0.7 ± 0.2	0.8 ± 0.2	**0.008**
**Moderate or severe AR**	31 (10.8%)	19 (10.5%)	12 (11.3%)	0.83
**Moderate or severe MR**	12 (4.2%)	8 (4.4%)	4 (3.8%)	0.79
**PASP, mmHg**	35.5 ± 19.4	35.7 ± 21.9	35.2 ± 15.5	0.94

Values are mean ± SD or n (%). AF = atrial fibrillation; AR= aortic regurgitation; BMI = body mass index; COPD= Chronic obstructive pulmonary disease; PCI=percutaneous coronary intervention; CABG=Coronary artery bypass graft; LVEF=left ventricular ejection fraction; AR = aortic regurgitation; HF = heart failure; MR= mitral regurgitation; NYHA= New York heart association; PCI= percutaneous coronary intervention; STS-PROM=Society of Thoracic Surgeons – predicted risk of mortality.

Table S2: Procedural characteristics According to the MI status (Q Wave MI vs Non-Q Wave MI) status [median follow-up =3.00 (1-4) years].

	Total	Non-Q Wave MI	Q Wave MI	p-value
	**N=287**	**N=181**	**N=106**	
**Valve-in-Valve procedure**	17 (5.9%)	13 (7.2%)	4 (3.8%)	0.24
**Approach**				**0.016**
**Transfemoral**	239 (83.3%)	159 (87.8%)	80 (75.5%)	
**Transcarotid**	14 (4.9%)	5 (2.8%)	9 (8.5%)	
**Sub-Clavicular**	34 (11.8%)	17 (9.4%)	17 (16.0%)	
**Secondary access**				0.67
**Femoral**	111 (39.9%)	69 (39.0%)	42 (41.6%)	
**Prosthesis type**				0.79
**Balloon-expandable**	1,649 (58.6%)	202 (59.2%)	1,447 (58.5%)	
**Self-expandable**	1,167 (41.4%)	139 (40.8%)	1,028 (41.5%)	
**Moderate or severe AR**	140 (5.9%)	31 (9.4%)	109 (5.3%)	0.003

Values are mean ± SD or n (%).

Table S3: 30-Day Clinical and Echocardiographic Outcomes According to the MI status (Q Wave MI vs Non-Q Wave MI).

	Total	Non-Q Wave MI	Q Wave MI	p-value
	**N=287**	**N=181**	**N=106**	
30-day outcomes				
**Death**	9 (3.1%)	7 (3.9%)	2 (1.9%)	0.35
**All Stroke**	7 (2.4%)	4 (2.2%)	3 (2.8%)	0.74
**Myocardial Infarction**	2 (0.7%)	0 (0.0%)	2 (1.9%)	0.064
**Major vascular complication**	27 (9.4%)	17 (9.4%)	10 (9.4%)	0.96
**Bleeding event according to VARC-3**				0.88
**Type 1**	20 (23.8%)	12 (22.6%)	8 (25.8%)	
**Type 2**	52 (61.9%)	33 (62.3%)	19 (61.3%)	
**Type 3**	11 (13.1%)	7 (13.2%)	4 (12.9%)	
**Type 4**	1 (1.2%)	1 (1.9%)	0 (0.0%)	
**Acute Kidney Injury**	25 (8.7%)	17 (9.4%)	8 (7.5%)	0.59
**Permanent pacemaker implantation**	57 (19.9%)	38 (21.0%)	19 (17.9%)	0.53
**Heart failure hospitalization**	3 (1.0 %)	1 (0.6%)	2 (1.9%)	0.61
Echocardiography post-procedure				
**LVEF, %**	43.3 ± 12.0	44.5 ± 11.5	40.3 ± 12.6	**0.028**
**Peak transaortic gradient, mmHg**	18.4 ± 8.3	18.5 ± 8.0	18.2 ± 8.8	0.82
**Mean transaortic gradient, mmHg**	9.8 ± 4.3	10.0 ± 4.2	9.6 ± 4.6	0.50
**Aortic valve area, cm^2**	1.8 ± 0.6	1.8 ± 0.6	1.8 ± 0.6	0.68
**Moderate or severe AR**	3 (1.0%)	1 (0.6%)	2 (1.9%)	0.28

Values are mean ± SD or n (%).

Table S4: Maximum follow-up Clinical Outcomes According to the MI status (Q Wave MI vs Non-Q Wave MI) [median follow-up =3.00 (1-4) years].

	Total	Non-Q Wave MI	Q Wave MI	p-value
	**N=287**	**N=181**	**N=106**	
**All Cause Death**	135 (47.0%)	83 (45.9%)	52 (49.1%)	0.60
**CV Death**	65 (48.1%)	40 (48.2%)	25 (48.1%)	0.99
**All Stroke**	23 (8.0%)	14 (7.7%)	9 (8.5%)	0.82
**Myocardial Infarction**	18 (6.3%)	6 (3.3%)	12 (11.3%)	**0.007**
**Endocarditis**	6 (2.1%)	4 (2.2%)	2 (1.9%)	0.85
**Any Hospitalization**	131 (45.6%)	83 (45.9%)	48 (45.3%)	0.93
**Heart failure Hospitalization (HFH)**	42 (14.6%)	25 (13.9%)	17 (16.1%)	0.55
**Combined death and /or HFH**	147 (51.2%)	89 (49.2%)	58 (54.8%)	0.35

Values are mean ± SD or n (%).

Table S5: Analysis of LVEF Recovery by Infarct Location (Anterior vs Non-Anterior Q-Wave MI)

	**Anterior Q-Wave MI**	**Non-Anterior Q Wave MI**	**p-value**
	**N=52**	**N=54**	
**Pre-TAVR LVEF**	36.0±10.4	36.5±10.0	0.79
**Post-TAVR LVEF**	40.0±13.0	42.6±12.1	0.30
**Δ LVEF (Post - Pre), %**	4.13±9.14	6.08±7.82	0.25

Values are mean ± SD or n (%). LVEF=left ventricular ejection fraction; TAVR=transcatheter aortic valve replacement; QWMI=Q-wave myocardial infarction; ΔLVEF=change in left ventricular ejection fraction (Post-TAVR minus Pre-TAVR);
